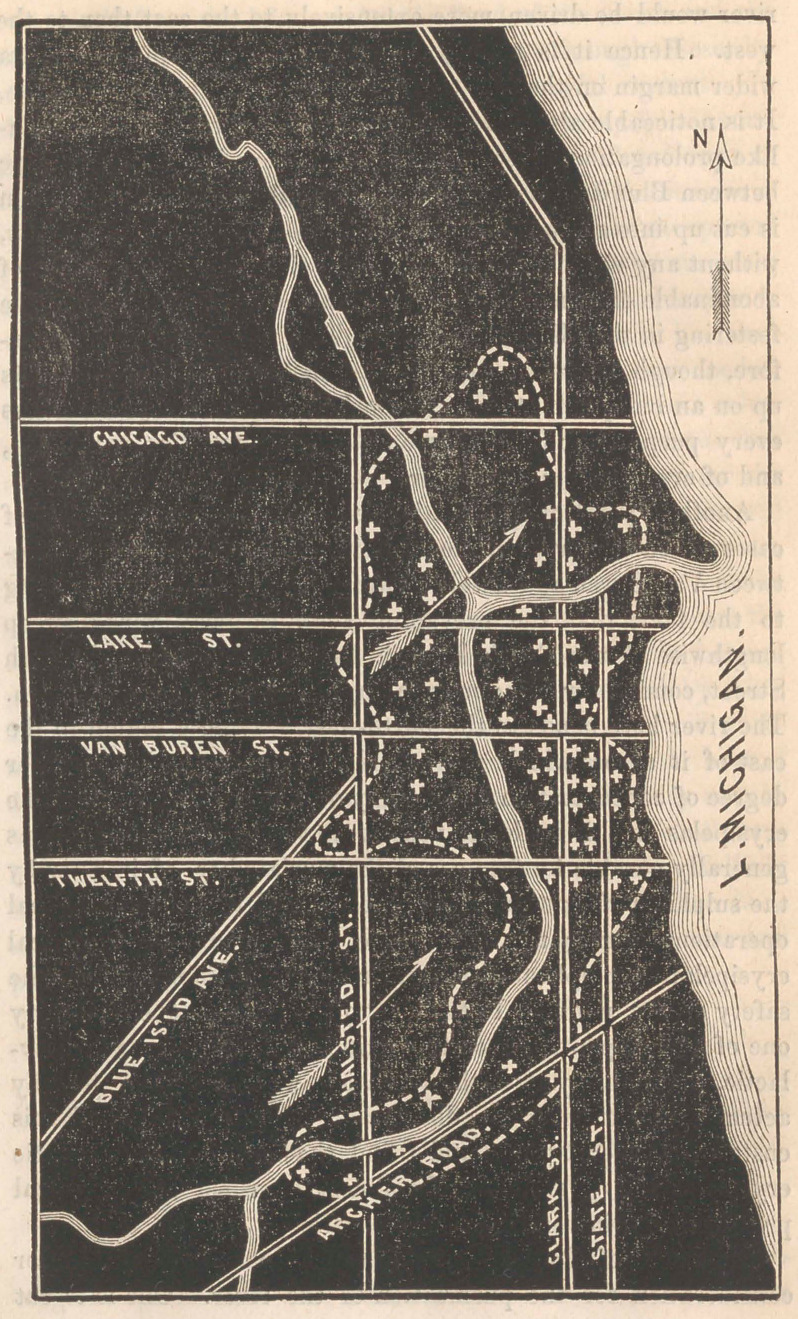# An Epidemic of Erysipelas, Caused by the Decomposition of Animal Matter in Chicago River

**Published:** 1864-01

**Authors:** E. Andrews

**Affiliations:** Professor of Surgery in Chicago Medical College


ARTICLE III.

### AN EPIDEMIC OF ERYSIPELAS, CAUSED BY THE DECOMPOSITION OF ANIMAL MATTER IN CHICAGO RIVER.

BY E. ANDREWS, M.D., Professor of Surgery in Chicago Medical College.

Chicago, like the city of London, has received a vast annoyance from the foul condition of its river; and the discussion as to what will be the best means of removing the evil is still on the tapis undecided. Chicago River, at ordinary times, has no proper current of its own. It is a mere inlet of Lake Michigan, receiving scarcely any water from inland sources, and having very little motion, except the slight flow inwards and outwards, caused by rise and fall of the lake, from changes of wind. By reference to the accompanying map it will be seen that it consists of a North and South Branch, each extending a few miles only, and uniting to form a short main river, three-quarters of a mile in length, terminating in the lake.

The river and its branches vary from 150 to 400 feet in width, and the depth is sufficient to float the largest lake vessels. Into this torpid stream, from the necessity of the case, nearly all the sewers of the city run, carrying the liquid filth from the streets, dwellings, sinks, manufactories, and slaughter houses of over 150,000 inhabitants. Along the South Branch in particular, there is a great number of packing houses, where many hundreds of thousands of animals are slaughtered every year, the filth and offal of which block up with a semi-fluid mass of putrefaction the sloughs and watercourses which empty into the river, while the more solid refuse materials are or were carried out and deposited in the fields beyond.

The rapid and enormous growth of the city gave to this evil such an unexpected magnitude that the sanitary precautions previously found sufficient, were now made utterly ineffective; and during the summer and autumn just passed, the condition and odor of the river became abominable beyond expression.

The stench could be perceived for miles in the direction of the wind; and the parts of the city nearest the stream were filled with the foul effluvium to an insupportable extent.

Now, there is nothing more thoroughly proved in the range of human knowledge than the fact, that foul air generates innumerable diseases. It is also a fact well known to physicians that one of the earliest effects of air, contaminated with putrifying matter, is the appearance of numerous cases of erysipelas among the persons exposed to its influence. In accordance with this general law of nature, the autumn of the past year showed a decided increase of sickness of such kinds as ordinarily scourge ill ventilated and filthy localities only, and that too in some of the best quarters of the city. Erysipelas in particular began to be very prevalent, and in the month of November, assumed the proportions of an epidemic. In order to decide the question of the influence of the river in this disease, I instituted inquiries among numerous physicians in all parts of the city, and obtained from them the localities of all the cases which had fallen under their care, during the months of October and November. The localities of all these cases amounting to 68 in all, are marked by white croses on the map, and the infected district thus determined is surrounded by a dotted line to show its form. The tract thus marked out contains only about one-third of the population of the city, yet it embraces almost every case of erysipelas that showed itself. The only apparent exceptions which I met were where persons were habitually doing business near the banks of the river and slept at a distance from it. Such persons would take the disease from the river air and then go home and be sick outside of the proper boundary of the epidemic, though they took the disease within it. A single glance at the map shows how thoroughly the epidemic clung to the vicinity of the river, and sufficiently demonstrates the cause of its inroads; but a few remarks may be needed to explain some peculiarities in the outline. The arrows mark the direction of the prevailing winds in this region. The current of air being more frequently from the Southwest than from any other quarter, it follows that the effluvium of the

N
CHICAGO AVE.
LAKE ST.
VAN BUREN ST.
TWELFTH ST.
BLUE IS'LD AVE.
HALSTED ST.
ARCHER ROAD.
CLARK ST.
STATE ST.
L. MICHIGAN.

river would be driven more extensively to the east than to the west. Hence it is readily observable that the epidemic had a wider margin on the east of the river than on the opposite side. It is noticeable also that the infected region had a peninsula-like prolongation westward, in spite of the wind, into the angle between Blue Island Avenue and Twelfth Street. This region is cut up into a great number of narrow and very filthy streets, without any efficient drainage. The slops, and every kind of abominable excrement, accumulate in the open gutters, and lie festering in the alleys and yards. This part of the city, therefore, though not receiving the full benefit of the river air, gets up on an independent basis a stench of its own, which answers every purpose for the increase of medical practice in general, and of erysipelas in particular.

Another point of interest is the enormous preponderence of cases which occurred on the east side of the South Branch, between Van Buren and Twelfth Streets. This is doubtless owing to the fact, that the prevailing south-westerly winds sweep lengthwise of the South Branch from Bridgeport to Twelfth Street, coming for three miles along a line of unspeakable filth. The river here bends to the north-west, leaving a region to the east of it across which the putrid effluvia sweep in a greater degree of concentration than in any other part of the city. The erysipelas in this epidemic usually attacked the face, and was generally curable by free use of the muriated tr. of iron, or by the sulphites of lime and soda. I continued to perform surgical operations during the whole time, without any of the fatal erysipelatus complications common in such epidemics, and the safety of my patients I attribute to the fact, that I put every one of them upon the use of the tincture of iron, as a prophylactic, immediately after the operation, without waiting for any actual appearance of the disease. I have now followed this custom for several years, and I find that in this way traumatic erysipelas and pyæmia may be almost banished from surgical practice, even in very unfavorable periods.

The Board of Public Works have had various plans under consideration for the purification of the river. One is to cut two canals from the lake, one to the head of the North and the other to the South Branch, and to throw in through these, by means of a wheel propelled by steam, a powerful current. In this way the water may be changed every twenty-four hours. Another plan is to cut the Illinois and Michigan Canal down to the level of the lake, and feed it by the river itself, thus drawing in pure water from the lake. The enormous and unexpected growth of the city, however, has taken everything by surprise; and before any plan could be executed, the evils of impurity were already upon us. At present, a temporary cure has been obtained by turning this way the waters of the Canal feeder, which create a current and keep the South Branch in a tolerably good condition. The kindly action of the frost too has covered the water with a thick stratum of ice, which is, for the winter, an effectual remedy. Next spring, however, the ice will melt, the canal feeder will be required for its original purposes, and, unless some effectual measures are taken, the evil will be upon us again with augmented magnitude. I have shown the effect of the river in only one disease, but it must not be supposed that erysipelas is the only sickness produced by filth. Typhoid and typhus fevers, malignant dysentery, typhoid pneumonia, and epidemic and malignant forms of a whole host of diseases follow in the train of such causes. No city can be bathed in putrid air with impunity; and if Chicago shall neglect to make a thorough removal of its filth, it will be infallibly scourged with these pestilences which through all time have smitten cities that commit similar sins against cleanliness and pure air.

## Figures and Tables

**Figure f1:**